# Responsiveness of PNPLA3 and lipid-related transcription factors is dependent upon fatty acid profile in primary bovine hepatocytes

**DOI:** 10.1038/s41598-021-04755-x

**Published:** 2022-01-18

**Authors:** Sophia J. Erb, Tawny L. Chandler, Heather M. White

**Affiliations:** 1 Department of Animal and Dairy Sciences, University of Wisconsin-Madison, 1675 Observatory Drive Rm 934B, Madison, WI 53706 USA; 2 Department of Population Medicine and Diagnostic Sciences, College of Veterinary Medicine, Cornell University, Ithaca, NY 14853 USA

**Keywords:** Lipids, Proteins, Cell biology, Mechanisms of disease

## Abstract

Knockdown of patatin-like phospholipase domain-containing protein 3 (PNPLA3) increased triglycerides (TG) in primary bovine hepatocytes, suggesting that PNPLA3 plays a causal role in hepatic TG clearing. In vivo, PNPLA3 abundance across the periparturient period is inversely related to hepatic TG accumulation and circulating fatty acid (FA) concentrations. The purpose of this research was to determine if PNPLA3, as well as other lipases, transcription factors, or FA-mediated genes, are regulated by FA mimicking liver lipid accumulation (ACCUM) and liver lipid clearing (RECOV) or singular FA physiologically found in dairy cows at 0.5 mM of circulating RECOV (iRECOV). Abundance of PNPLA3 tended to decrease with ACCUM and increased quadratically with RECOV (*P* ≤ 0.10), differing from *PNPLA3* expression, but consistent with previous in vivo research. Adipose TG lipase abundance, but not other lipase abundances, was quadratically responsive to both ACCUM and RECOV (*P* ≤ 0.005). Abundance of PNPLA3 and SREBP1c and expression of *LXRA* responded similarly to iRECOV, with C18:0 tending to decrease abundance (*P* ≤ 0.07). Results indicate that bovine PNPLA3 is translationally regulated by FA and although a *LXRA*-SREBP1c pathway mediation is possible, the mechanism warrants further investigation.

## Introduction

It is well established in the dairy industry that fatty liver syndrome (FLS) occurs during the periparturient period in dairy cows^1^. Fatty acids (FA) are mobilized from adipose tissue via lipolysis and circulate in the bloodstream to the liver, where they are taken up for various fates including re-esterification as triglycerides (TG) for storage within hepatocytes, resulting in the onset of FLS^2–4^. Monetary losses of FLS were estimated to be $60 million in 2004^5^ and likely exceed that now. While FLS remains a hinderance to cow productivity, the molecular underpinnings of FLS in dairy cows and how to mitigate it remain unknown.

Many aspects of lipid metabolism are conserved across species, and research focusing on nonalcoholic fatty liver disease (NAFLD) and steatohepatitis in humans may provide insight into the etiology of FLS in dairy cattle and vice versa. While there are a variety of differences between ruminants and nonruminants regarding physiology and digestive capabilities^6–8^, a key lipase involved in the development of NAFLD also plays a critical and similar role in FLS. In humans, a single nucleotide polymorphism (SNP) inactivates the lipase patatin-like phospholipase domain-containing protein 3 (PNPLA3), resulting in TG accumulation and storage in the liver^9–11^. An in vivo study elucidated that periparturient dairy cows with more hepatic PNPLA3 abundance had lower hepatic TG^12^. In conjunction with those findings, knockdown of PNPLA3 abundance using siRNA in primary bovine hepatocytes resulted in greater cellular TG content compared to cells treated with a nonspecific siRNA sequence^12^. These patterns are consistent with the naturally occurring inactivating SNP in humans; however, a similar pattern was not observed in mice, where knockout of PNPLA3 did not affect hepatic TG content^13^.

Despite the causal role of PNPLA3 in hepatic TG accumulation in both humans and bovine, the transcriptional and translational regulation of PNPLA3 remains elusive. Additionally, an understanding of the coordinated response of PNPLA3 with other lipid-related proteins is needed. Based on in vivo data, our hypothesis is that bovine PNPLA3 will be responsive to FA in a concentration and composition dependent manner, with FA composition and concentration associated with increased PNPLA3 abundance, and that this regulation may be transcription factor (TF) mediated. Thus, the objectives of this study were to 1) determine how PNPLA3 responds to FA presented in mixtures representing two distinct phases of the periparturient period [period of liver lipid accumulation (ACCUM) or period of liver lipid clearing (RECOV)], 2) determine if potential responses to FA mixtures can be attributed to individual FA [concentration of individual FA physiologically found in dairy cows present in circulating serum at 0.5 mM (iRECOV)], and 3) determine potential regulation of PNPLA3 through a targeted analysis of genes and proteins associated with lipolysis and FA-mediation.

## Results

### PNPLA3 cellular localization, mRNA expression, and protein abundance

**Figure 1:**
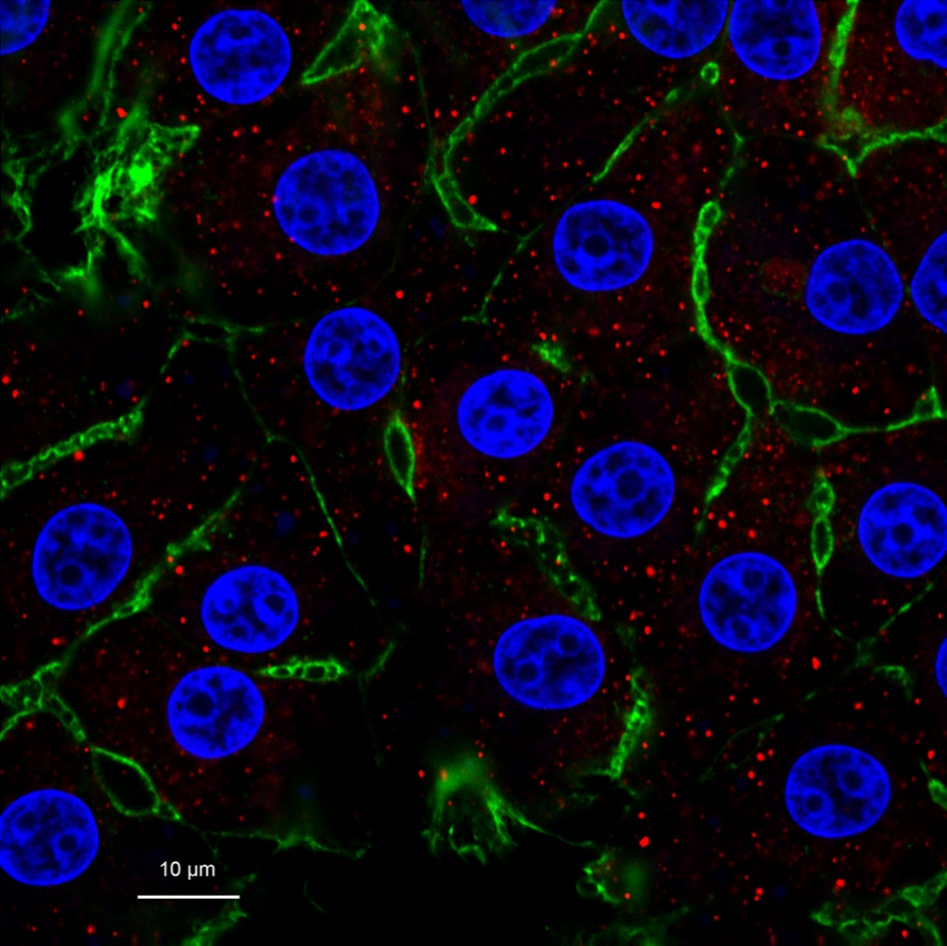
Primary bovine hepatocytes stained with DAPI (nuclei; blue), Alexa Fluor 488 Phalloidin (F-actin; green), and Cy3 for patatin-like phospholipase domain-containing protein 3 (PNPLA3; red). A common trait in hepatocytes, some hepatocytes display binucleated nuclei and some do not. The cytoskeletal protein F-actin outlines the shape of the cells. Appearance of PNPLA3 in red shows it scattered throughout the cytoplasm of the cells, where lipolytic activity occurs.

**Figure 2:**
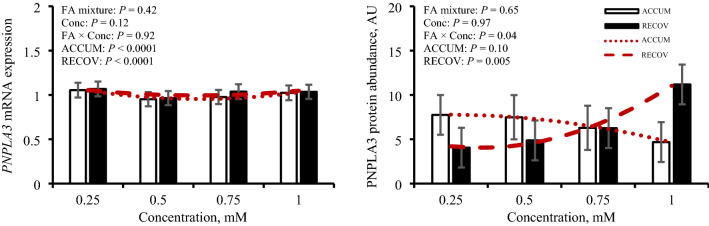
Gene expression and protein abundance of patatin-like phospholipase domain-containing protein 3 (PNPLA3) in response to fatty acid (FA) mixture representing liver lipid accumulation (ACCUM; open bars and dotted line) and fatty acid mixture representing liver lipid clearing (RECOV; closed bars and dashed line) at different concentrations in primary bovine hepatocytes. Data is presented as least squares mean ± standard error of the means. Quadratic relationships are displayed if *P* ≤ 0.15 for a main effect.

Stained primary bovine hepatocytes (Fig. 1) indicate scattered cytoplasmic localization of PNPLA3 protein within the hepatocytes, consistent with location of lipolytic activity and lipid droplets. Expression of *PNPLA3* and its resulting protein abundance is in Fig. 2. An opposing quadratic relationship existed for *PNPLA3* expression with ACCUM (*P* < 0.0001) and RECOV (*P* < 0.0001). Abundance of PNPLA3 was affected (*P* = 0.04) by concentration × FA mixture. A quadratic relationship was observed where abundance tended to decrease (*P* = 0.10) quadratically with greater concentrations of ACCUM, whereas PNPLA3 abundance was increased (*P* = 0.005) quadratically with higher concentrations of RECOV. No change in *PNPLA3* expression was observed with iRECOV treatment (*P* = 0.48; Table 2). Conversely, abundance of PNPLA3 was affected by iRECOV (*P* = 0.03; Fig. 7) and tended to decrease (*P* = 0.07) with C18:0 compared to C18:2 n-6, and did decrease with C18:0 compared to C22:6 n-3 (*P* = 0.05; Fig. 7).

### Protein abundance of lipases and gene expression of FA-mediated genes and TFs

**Figure 3:**
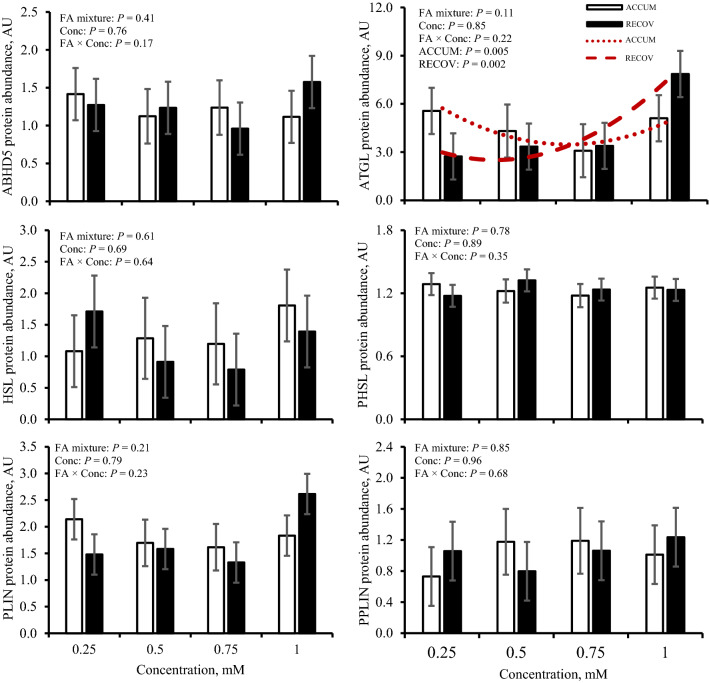
Protein abundance of abhydrolase domain containing 5 (ABHD5), adipose triglyceride lipase (ATGL), hormone sensitive lipase (HSL), phosphorylated HSL (PHSL), perilipin (PLIN), and phosphorylated PLIN (PPLIN) in response to fatty acid mixture representing liver lipid accumulation (ACCUM; open bars and dotted line) and fatty acid mixture representing liver lipid clearing (RECOV; closed bars and dashed line) at different concentrations in primary bovine hepatocytes. Data is presented as least squares mean ± standard error of the means. Quadratic relationships are displayed if *P* ≤ 0.15 for a main effect.

An opposing quadratic relationship for adipose TG lipase (ATGL) existed in which ATGL decreased with ACCUM (*P* = 0.005) and increased with RECOV (*P* = 0.002; Fig. 3). No change was observed on abundance of abhydrolase domain containing 5 (ABHD5), hormone sensitive lipase (HSL), phosphorylated HSL (PHSL), perilipin (PLIN), and phosphorylated PLIN (PPLIN) in response to FA mixtures at varying concentrations (*P* ≥ 0.17; Fig. 3).

**Figure 4:**
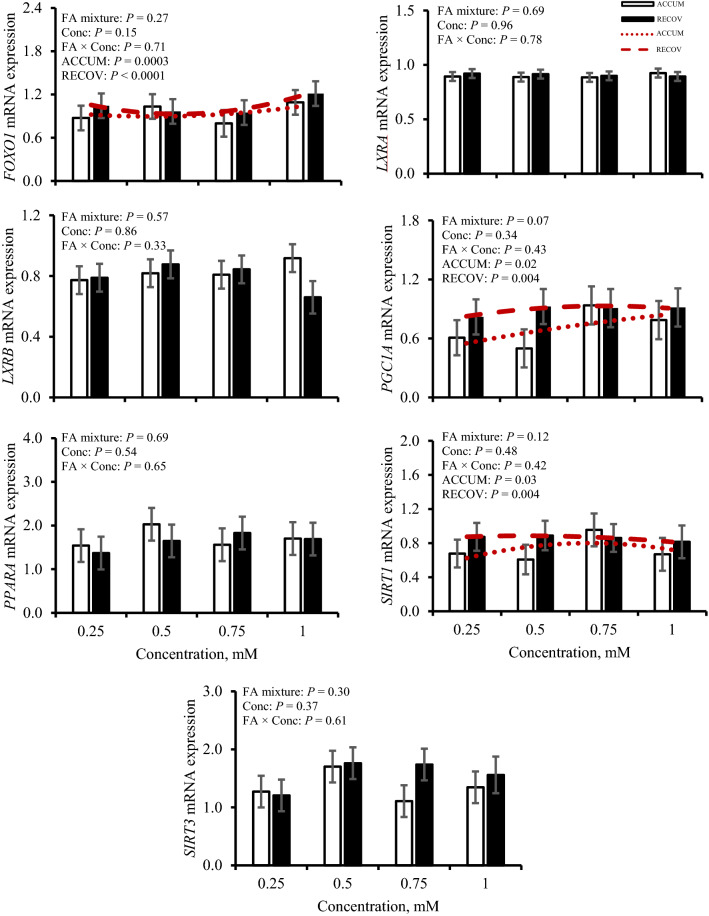
Gene expression of transcription factors forkhead box O1 (*FOXO1*), liver X receptor α (*LXRA*), liver X receptor β (*LXRB*), peroxisome proliferator-activated receptor gamma coactivator 1-alpha (*PGC1A*), peroxisome proliferator-activated receptor α (*PPARA*), sirtuin 1 (*SIRT1*), and sirtuin 3 (*SIRT3*) in response to fatty acid mixture representing liver lipid accumulation (ACCUM; open bars and dotted line) and fatty acid mixture representing liver lipid clearing (RECOV; closed bars and dashed line) at different concentrations in primary bovine hepatocytes. Data is presented as least squares mean ± standard error of the means. Quadratic relationships are displayed if *P* ≤ 0.15 for a main effect.

**Figure 5:**
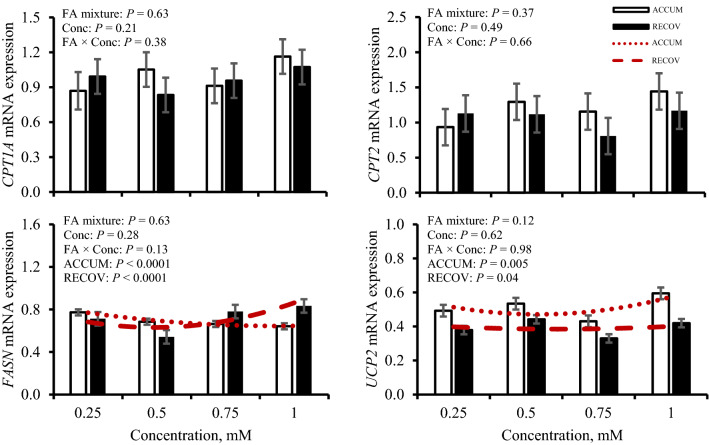
Gene expression of fatty acid-mediated genes carnitine palmitoyltransferase 1A (*CPT1A*), carnitine palmitoyltransferase 2 (*CPT2*), fatty acid synthase (*FASN*), and uncoupling protein 2 (*UCP2*) in response to fatty acid mixture representing liver lipid accumulation (ACCUM; open bars and dotted line) and fatty acid mixture representing liver lipid clearing (RECOV; closed bars and dashed line) at different concentrations in primary bovine hepatocytes. Data is presented as least squares mean ± standard error of the means. Quadratic relationships are displayed if *P* ≤ 0.15 for a main effect.

Responses of expression of FA-mediated genes and TFs regarding FA mixture and concentration treatment are in Figs. 4 and 5, respectively, and responses to iRECOV are in Table 2. Expression of FA-transport genes carnitine palmitoyltransferase 1A (*CPT1A*), carnitine palmitoyltransferase 2 (*CPT2*), and peroxisome proliferator-activator α (*PPARA*) were not altered by FA mixture nor concentration (*P* ≥ 0.21). However, *CPT2* expression tended to change with treatment of iRECOV (*P* = 0.08). Neither *CPT1A* nor *PPARA* were affected by iRECOV (*P* ≥ 0.21). An opposing quadratic relationship of fatty acid synthase (*FASN*) expression was observed with ACCUM (*P* < 0.0001) and RECOV (*P* < 0.0001); however, *FASN* expression was not altered by iRECOV (*P* = 0.87). A quadratic relationship was observed for sirtuin 1 (*SIRT1*) expression to change with both ACCUM (*P* = 0.03) and RECOV (*P* = 0.004), yet expression was not altered by iRECOV (*P* = 0.60). Expression of sirtuin 3 (*SIRT3*) was not altered by any FA treatment (*P* ≥ 0.30). A quadratic relationship was observed for uncoupling protein 2 (*UCP2*) expression in response to both ACCUM (*P* = 0.005) and RECOV (*P* = 0.04), yet expression was not altered by iRECOV (*P* = 0.60).

Expression of liver X receptor α (*LXRA*) and liver X receptor β (*LXRB*) were not affected by FA mixture nor concentration (*P* ≥ 0.33); however, expression of *LXRA* was affected by iRECOV (*P* = 0.03). Expression decreased (*P* = 0.03) with C18:0 compared to C14:0 and tended to decrease (*P* = 0.06) with C16:0 compared to C14:0. No evidence was observed for *LXRB* to alter expression in response to iRECOV (*P* = 0.30). Expression of peroxisome proliferator- activated receptor gamma coactivator 1 α (*PGC1A*) tended to be affected by FA mixture (*P* = 0.07); expression increased quadratically with increasing concentration of ACCUM (*P* = 0.02) and RECOV (*P* = 0.004), but expression was not affected by iRECOV (*P* = 0.87). A quadratic relationship for forkhead box O1 (*FOXO1*) expression existed with ACCUM (*P* = 0.003) and RECOV (*P* < 0.0001) but expression was not affected by iRECOV (*P* = 0.33).

### ChREBP and SREBP1c mRNA expression and protein abundance

**Figure 6:**
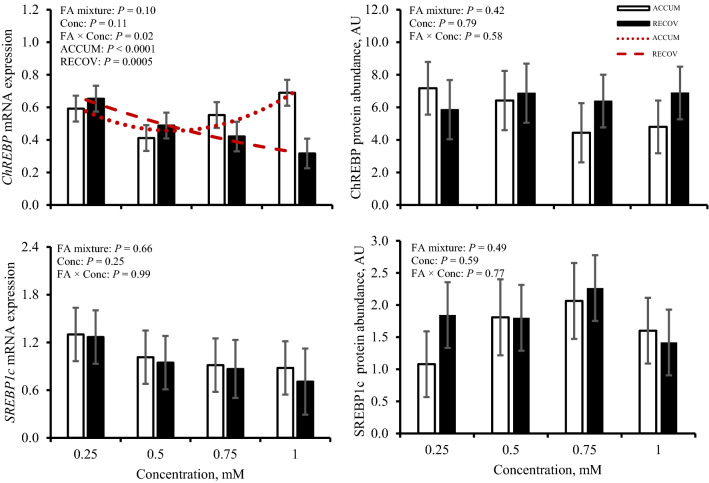
Gene expression and protein abundance of carbohydrate response element binding protein (ChREBP) and sterol regulatory element-binding transcription factor 1c (SREBP1c) in response to fatty acid mixture representing liver lipid accumulation (ACCUM; open bars and dotted line) and fatty acid mixture representing liver lipid clearing (RECOV; closed bars and dashed line) at different concentrations in primary bovine hepatocytes. Data is presented as least squares mean ± standard error of the means. Quadratic relationships are displayed if *P* ≤ 0.15 for a main effect.

**Figure 7:**
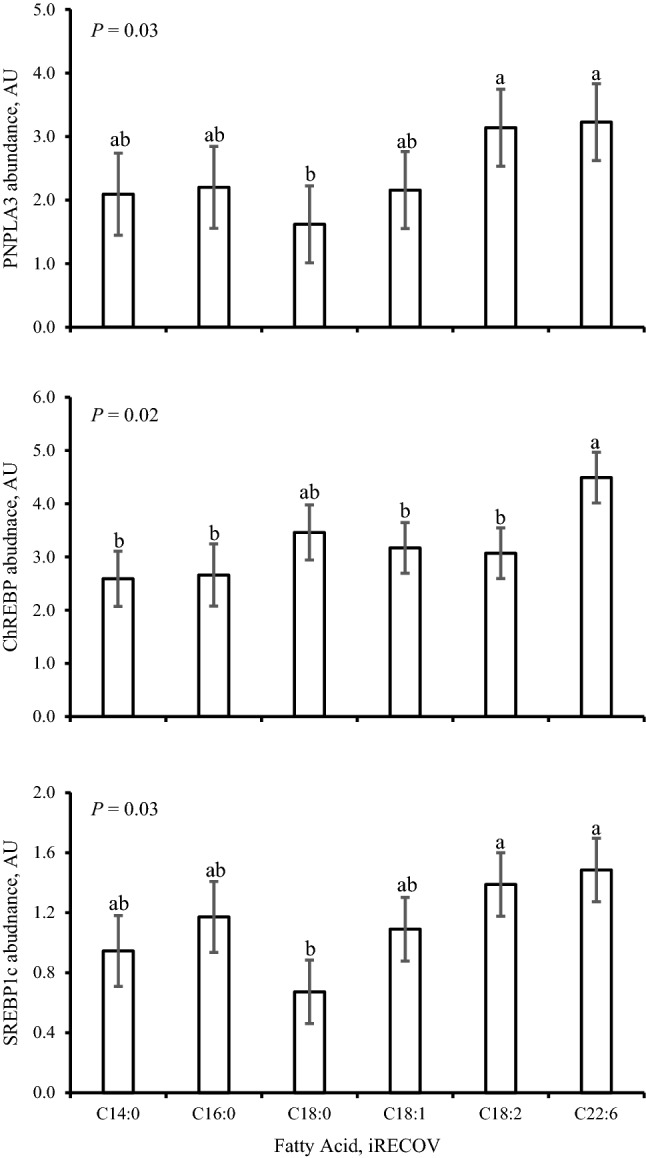
Protein abundance of patatin-like phospholipase domain-containing protein 3 (PNPLA3), carbohydrate response element binding protein (ChREBP), and sterol regulatory element-binding transcription factor 1c (SREBP1c) in response to individual fatty acids at the concentration physiologically found circulating in serum in dairy cows at 0.5 mM of circulating liver lipid clearing profile (iRECOV) in primary bovine hepatocytes. C14:0, C16:0, C18:0, C18:1 n-9, C18:2 n-6, and C22:6 n-3 are abbreviated as C14:0, C16:0, C18:0, C18:1, C18:2, and C22:6 in the figure for conciseness. Data is presented as least squares mean ± standard error of the means. Differences between individual fatty acids are indicated as alphabetic superscripts; when superscripts do not share the same letter, differences between the fatty acids were found (*P* ≤ 0.07).

Expression of carbohydrate response element binding protein (*ChREBP*) and sterol regulatory element-binding transcription factor 1c (*SREBP1c*) and resulting protein abundances are shown in Fig. 6. Expression of *ChREBP* was affected (*P* = 0.02) by concentration × FA mixture. A quadratic relationship was observed where expression was affected (*P* < 0.0001) by greater concentrations of ACCUM, whereas with higher concentrations of RECOV, *ChREBP* expression was quadratically increased (*P* = 0.0005). No evidence was observed for *ChREBP* expression to be altered with iRECOV (*P* = 0.85). Abundance of ChREBP was not affected (*P* ≥ 0.42) by concentration nor FA mixture, but ChREBP abundance was affected by iRECOV (*P* = 0.02; Fig. 7). Abundance of ChREBP tended to decrease with C18:0 (*P* = 0.08) and C18:2 n-6 (*P* = 0.06) compared to C22:6 n-3 and did decrease (*P* ≤ 0.05) with C14:0 and C16:0 compared to C22:6 n-3.

No evidence was observed for expression of *SREBP1c* to be affected by concentration or FA mixture (*P* ≥ 0.25). Expression of *SREBP1c* was not affected by iRECOV (*P* = 0.34). Abundance of SREBP1c was not affected (*P* ≥ 0.49) by concentration nor FA mixture, but SREBP1c abundance was affected by iRECOV (*P* = 0.03; Fig. 7). Abundance of SREBP1c tended to decrease with C18:0 (*P* = 0.06) compared to C18:2 n-6 and did decrease (*P* = 0.03) with C18:0 compared to C22:6 n-3.

## Discussion

Understanding the etiology of the development and subsequent recovery of FLS in periparturient dairy cows is key to mitigating FLS and may provide mechanistic insights into understanding similar metabolic disorders in other species. Administering FA mixtures mimicking the physiological states during FLS development and determining if individual FA within the mixture is responsible for regulating a wide variety of genes and proteins involved in lipolysis, FA-transport, or are FA-mediated is a key piece of furthering our understanding. Therefore, the primary objective was understanding the regulation of PNPLA3 due to its impact on FLS in dairy cows. In humans and mice, PNPLA3 is localized in the cytoplasm and on the lipid droplet^9,14,15^. Likewise, bovine PNPLA3 appears to be localized in a similar pattern (Fig. 1). Expression of *PNPLA3* and its resulting protein abundance did not exhibit similar responses, suggesting that regulation, at least in response to FA, is primarily at the translational level.

To elucidate which singular FA was responsible for this shift, iRECOV treatments were administered. Given the opposing quadradic response of PNPLA3 abundance to increasing concentrations RECOV and ACCUM (Fig. 2), individual FA most likely to be responsible for the regulation would be those with differential inclusion proportions between the two physiological mixtures, specifically C18:0, C18:1 n-9, or C20:5 n-3 (Table 1). In bovine, C18:0 originates from rumen biohydrogenation of dietary polyunsaturated FA and adipose tissue stores^16,17^, making it a key component of circulating FA profile during mobilization (ie, the physiological phase represented by ACCUM). C18:0 can also be stored in hepatocytes^16,17^ and thus could potentially be released from hepatic TG by lipolysis during periods of liver TG recovery (ie. the physiological phase represented by RECOV). Treatment with C18:0 inhibited PNPLA3 when presented alone; however, greater contribution of C18:0 to the RECOV mixture would imply that increasing concentration of that mixture would decrease PNPLA3, which was not observed. Although C18:0 has been demonstrated to have inhibitory regulatory effects in isolation, these effects are often blighted when cells are treated in mixtures that reflect physiological states^18,19^. In contrast, C18:1 n-9 and C18:2 n-6 are found in lower concentration in RECOV than ACCUM and both resulted in increased PNPLA3 abundance compared with C18:0 and maintained abundance compared with C14:0 and C16:0 (Fig. 7). These findings suggest that C18:1 n-9 and C18:2 n-6 could be inhibiting PNPLA3 abundance, as evidenced by the decreased abundance during treatment with ACCUM but increased abundance with RECOV (containing less of these FA) and exposure to iRECOV. This is in agreement with human hepatocytes treated with C18:1 n-9 and C18:2 n-6, where PNPLA3 abundance increased, even though treatment was not at a physiologically relevant concentration (400 µM)^20^. Since hepatocytes are never exposed to single fatty acids in vivo, it is not surprising that the combination of FA is an important component of regulation. Ultimately, the pattern observed within is in agreement with prior in vivo work^12^.

**Table 1:** Treatment composition of fatty acids (FA) of physiologically representative mixtures present in circulating serum [liver lipid accumulation (ACCUM) and liver lipid clearing (RECOV)] and concentration of individual FA physiologically present in circulating serum in dairy cows at 0.5 mM of RECOV (iRECOV).

	FA^a^	Mixture composition, %		iRECOV, mM
		ACCUM	RECOV	
Myristic	C14:0	2.63	1.81	0.009
Palmitic	C16:0	33.01	34.93	0.175
Stearic	C18:0	43.39	55.81	0.279
Oleic	C18:1	16.4	4.2	0.021
Linoleic	C18:2	1.3	0.95	0.005
EPA	C20:5	0.9	–	–
DHA	C22:6	2.38	2.26	0.011

^a^C14:0, C16:0, C18:0, C18:1 n-9, C18:2 n-6, C20:5 n-3, and C22:6 n-3 are abbreviated as C14:0, C16:0, C18:0, C18:1, C18:2, C20:5 and C22:6 in the table for conciseness.

Expression of *PNPLA3* and abundance of PNPLA3 were not congruent in this in vitro study, which is in agreement with previous findings^12^. It may be possible that the differences seen in *PNPLA3* expression and resulting protein both in vitro and in vivo are due to regulation at both the transcriptional and translational levels. Energy status is known to affect PNPLA3 protein in rodents and humans^20^, and a growing body of evidence is in agreement that bovine *PNPLA3* mRNA and its resulting protein are also affected by energy status^12,21^. Although cell culture models cannot fully recapitulate physiological energy status, a recent in vivo study demonstrated the relationships between energy status, liver TG, and PNPLA3 protein abundance^12^. Given that hepatic TG accumulation is responsive to energy status during the periparturient period, the lower abundance of PNPLA3 in this study in response to ACCUM, yet higher abundance in response to RECOV, further supports the importance of PNPLA3 abundance on clearing TG in vivo and *in vitro*^12^.

The TF SREBP1c is a known regulator of lipogenesis^22^ and growing evidence suggests SREBP1c may play a role in NAFLD^23^. Abundance of SREBP1c protein responded similarly to iRECOV as PNPLA3 protein abundance did (Fig. 7). Both PNPLA3 and SREBP1c were decreased with C18:0 compared to C22:6 n-3, while ChREBP protein abundance was not affected by C18:0. The findings presented here suggest that transcriptional and translational levels of bovine PNPLA3 are differentially regulated, and similar to human PNPLA3, bovine PNPLA3 protein may be regulated by SREBP1c^20,24,25^ although further research is needed to elucidate the direct role of SREBP1c on PNPLA3 abundance in bovine.

Although the rate limiting lipase, ATGL decreased quadratically with ACCUM and increased quadratically with RECOV, its coactivtor, ABHD5^26^, was not affected by FA mixture nor concentration. This finding suggests that the role ATGL has in FLS within hepatocytes may not be as prominent as the role it has in lipolysis in adipose tissue^27–29^. This is further supported by the lack of change we observed in hepatocyte ATGL abundance during PNPLA3 knockdown in bovine hepatocytes^12^. Even in rodents, knockout of ATGL in hepatocytes reduces, but does not dissipate, TG hydrolysis because other lipases compensate for the lack of ATGL^29^. Across species, the working hypothesis is that PLIN encapsulates the lipid droplet and when the AMPK pathway is activated, both HSL and PLIN are phosphorylated, allowing ATGL, ABHD5, and PHSL access to the lipid droplet^26,28,30^. Previous work focused on lipolysis in adipose tissue in dairy cows found that ATGL, PHSL, and PPLIN abundance were affected by the periparturient period but ABHD5, HSL, and PLIN were not^28^. This further implicates the role PNPLA3 has on FLS.

Across species, *CPT1A* is regulated by *PPARA* and transports FA into the mitochondria^31^ followed by transport into the mitochondrial matrix for β-oxidation by *CPT2,* which was recently identified as the rate-limiting step of β-oxidation in cancerous human cell lines^32,33^. Multiple bovine studies have conflicting evidence on if *CPT1A* expression varies over the periparturient period^34^ or remains constant^31,35^, where these studies focus on various treatment effects (i.e., excess dietary energy, nutritional intervention, etc.) and the effect of time on expression. In the current work, there was no response of *CPT1A* and *CPT2* to ACCUM nor RECOV, although *CPT2* tended to be responsive to iRECOV (Table 2). Previous work in dairy cows found that both *CPT1A* and *CPT2* expression were both lower with hyperketonemia, a common peripartum metabolic disorder defined as high concentrations (≥ 1.2 mM) of circulating β-hydroxybutyrate, but expression was only measured at one time point during the study^36^. The lack of consistent evidence in the literature prevents us from drawing conclusions on *CPT* expression responsiveness in bovine.

**Table 2:** Least squares means and standard error of the means (SE) of the gene expression of genes of interest in primary bovine hepatocytes treated with individual fatty acids physiologically found circulating in dairy cows at an in vivo mixture at 0.5 mM^1^ during liver lipid clearing (iRECOV) with significant differences between means denoted with different superscript letters.

Gene	iRECOV^2^						SE	*P*-value
	C14:0	C16:0	C18:0	C18:1	C18:2	C22:6		
*ChREBP*	0.91	0.89	0.88	0.91	0.90	0.90	0.04	0.85
*CPT1A*	0.80	1.11	0.70	0.78	0.52	0.80	0.27	0.64
*CPT2*	0.98	0.97	0.98	0.89	0.96	0.98	0.05	0.08
*FASN*	0.75	0.67	0.66	0.69	0.73	0.75	0.10	0.87
*FOXO1*	1.05	0.89	0.89	1.00	1.08	0.89	0.09	0.33
*LXRA*	0.80^a^	0.60^b^	0.57^b^	0.72^ab^	0.68^ab^	0.73^ab^	0.10	0.03
*LXRB*	0.97	0.94	0.92	0.86	0.54	0.64	0.19	0.30
*PGC1A*	0.93	0.94	0.95	0.95	0.95	0.95	0.03	0.87
*PNPLA3*	0.99	0.99	1.04	1.01	1.06	1.06	0.07	0.48
*PPARA*	0.95	0.95	1.00	1.00	1.00	0.91	0.07	0.21
*SIRT1*	0.90	0.91	0.93	0.94	0.94	0.93	0.03	0.60
*SIRT3*	0.40	0.23	0.21	0.29	0.18	0.37	0.12	0.44
*SREBP1*	1.94	2.60	2.27	1.84	0.84	1.49	0.62	0.34
*UCP2*	0.40	0.23	0.21	0.29	0.18	0.37	0.19	0.60

^1^Concentratoin of C14:0, C16:0, C18:0, C18:1 n-9, C18:2 n-6, and C22:6 n-3 was treated at 0.009, 0.175, 0.279, 0.021, and 0.011 mM, respectively.

^2^C14:0, C16:0, C18:0, C18:1 n-9, C18:2 n-6, C20:5 n-3, and C22:6 n-3 are abbreviated as C14:0, C16:0, C18:0, C18:1, C18:2, C20:5 and C22:6 in the table for conciseness.

Sirtuins are a family of genes involved in deacetylation that are well conserved across species^37^. Divided into 4 classes, *SIRT1* and *SIRT3* are categorized into class I, which primarily encompasses involvement in metabolic diseases and FA oxidation^37^. While *SIRT1* is located in the nucleus and cytoplasm, the location of *SIRT3* is in the mitochondria and cytoplasm^37,38^. A previous study in which dairy cows were identified as having FLS (defined as hepatic TG content of ≥ 5% wet weight) had lower *SIRT3* expression in response to higher concentrations of circulating FA^39^. While this is a similar pattern observed in mice^40^, these findings are contrary to ours. It should be noted that *SIRT3* can regulate *PPARA* in humans^38^. Both *SIRT1* and *PGC1A* exhibited similar patterns of expression with administration of FA mixture and concentration. In human pluripotent stem cells, *SIRT1* knockdown significantly decreased *PGC1A*, resulting in a decrease in lipolysis^41^. Previous work in mice determined that *SIRT1* successfully induced complete FA oxidation via deacetylation of PGC1A with treatment of C18:1 n-9^42^ and that deacetylation of PGC1A increases gluconeogenesis^43^. However, with *PGC1A* expression tending to be greater with RECOV, it is possible that this TF may regulate PNPLA3 protein in FLS (Fig. 4). Evidence in humans suggests that SIRT1-mediated pathways aided in resolving NAFLD by increasing NAD + in the liver, but knockdown of *SIRT3* does not change liver status^38^. With *SIRT1* and *PGC1A* having a quadratic response to ACCUM and RECOV but *SIRT3* and *PPARA* having no response, it may be possible that the SIRT1-mediated pathway may aid in resolving FLS in bovine. More work on clarifying the relationships between *SIRT1*, *PGC1A*, and *PNPLA3* mRNA and its resulting protein in FLS and primary bovine hepatocytes is warranted^29,42,44^.

The TF *FOXO1* plays a role in insulin signaling, regulates lipid metabolism and gluconeogenesis in NAFLD patients, and regulates *SREBP1c* in mice^45^. Expression of both *FOXO1* (Fig. 4) and *UCP2* (Fig. 5), a gene involved in insulin signaling, had a quadratic relationship with ACCUM and RECOV, but the pattern of expression of both genes differed with RECOV. In vitro mouse adipocyte research observed that lower *FOXO1* expression appeared to cause lower *UCP2* expression^46^, and that in goat mammary epithelial cells, knockdown of *FOXO1* increased both *FASN* expression and TG synthesis^47^. Fatty acid synthase is key in synthesizing FA, promotes lipid storage, and has previously been shown to be upregulated in NAFLD patients^48^. In our study, the quadratic relationship of *FASN* expression increased with RECOV, although *FASN* was not responsive to individual FA. Expression of *PNPLA3* (Fig. 2) and of *FOXO1* (Fig. 4) did not exhibit the same expression patterns with FA mixtures across concentrations. The relationships between these three genes and proteins are unclear and should be further explored.

Transcription factor *LXRA* regulates biochemical pathways involved in regulating inflammatory responses, glycolysis, and lipid metabolism^22,49^ with primary action in the liver, compared to the isoform *LXRB* which is ubiquitously expressed in tissues across species^49,50^. Downstream targets of *LXRA* are *ChREBP* and *SREBP1c*, both regulators of glycolysis and lipogenesis, respectively^22,48^, and known regulators of PNPLA3 in mice and humans. Surprisingly, although *LXRA* expression was responsive to iRECOV, *ChREBP* and *SREBP1c* expression were not; however, *LXRA* expression and SREBP1c abundance both decreased with C18:0 iRECOV treatment (Table 2, Fig. 7). Since *LXRA* expression, SREBP1c abundance, and PNPLA3 abundance exhibited similar patterns in response to iRECOV, this finding may further provide evidence that the *LXRA*-SREBP1c pathway is regulating PNPLA3 abundance.

## Conclusions

**Figure 8:**
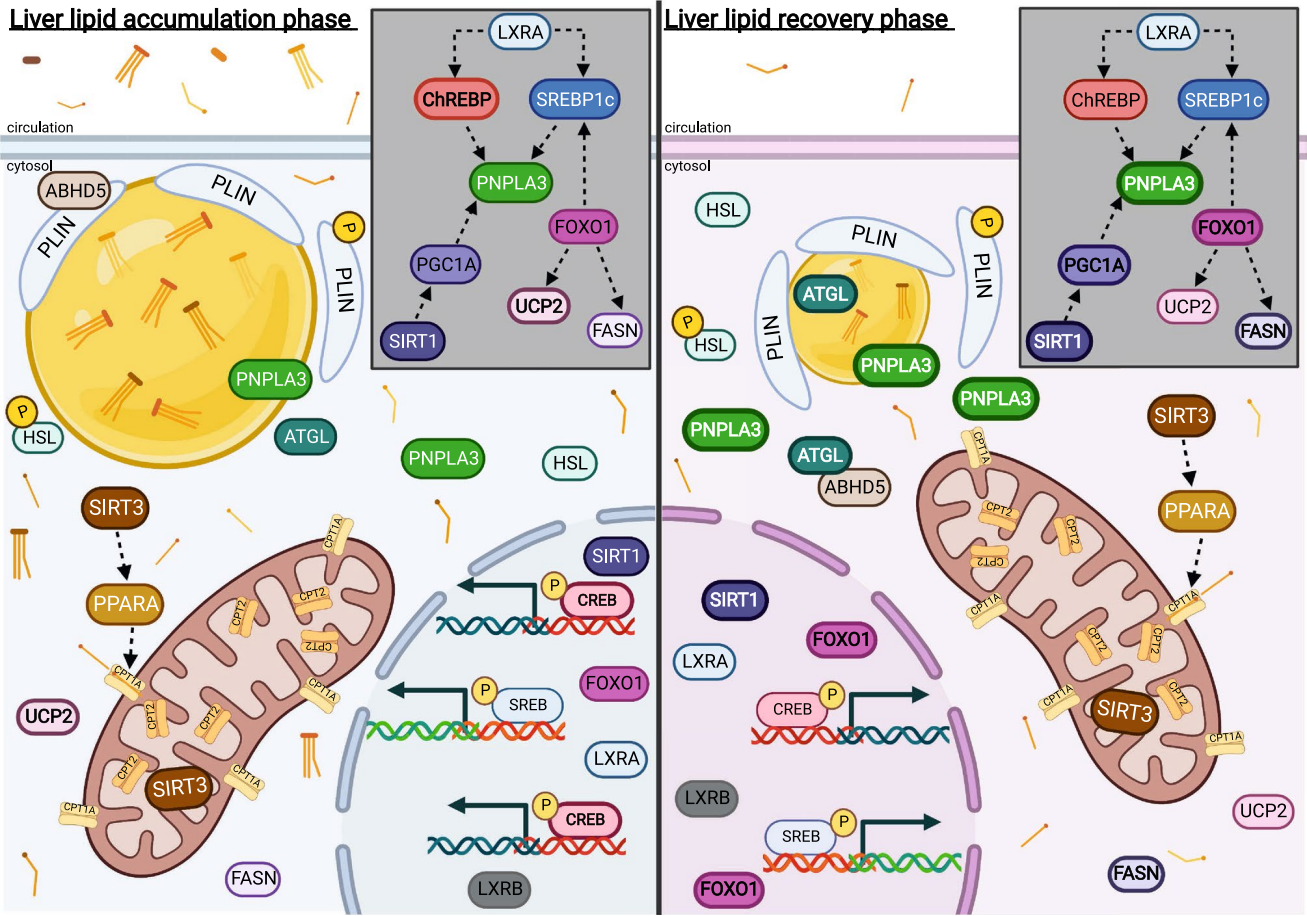
Working hypothesis of lipid related genes and proteins in primary bovine hepatocytes during the liver lipid accumulation phase (left panel) and the liver lipid recovery phase (right panel). The gray box insert within each panel represents the associations between PNPLA3, transcription factors, and genes where potential for regulatory influence exists. Relative abundance between panels are shown visually with line weight and quantity. Arrows between genes and proteins indicate associative relationships based on published research across species and the current data and do not denote directionality of regulation. Created with BioRendeder.com.

Bovine PNPLA3 appears to be differentially regulated at the transcriptional and translational level, and the importance of mimicking physiological states via FA mixtures rather than singular FA provides a more complete picture of regulation. Although bovine PNPLA3 abundance is clearly responsive to FA in a manner that is consistent between primary bovine hepatocytes herein, and previously published in vivo research, the relationships between the gene expression of *SIRT1*, *PGC1A*, *SIRT3*, *PPARA*, and *PNPLA3* and its resulting protein remain unclear and warrant further investigation into which pathway aids in FLS recovery. A working hypothesis of associations between these key components is shown in Fig. 8, based on published research across species and the current data. Based on the current work, it is possible that PNPLA3 regulation is mediated by SREBP1c via *LXRA*, similar to PNPLA3 regulation in humans, due to the similar pattern of response to iRECOV. Further research elucidating the role of SREBP1c and *LXRA* on bovine PNPLA3 regulation could provide valuable insight leading to both bovine targeted interventions and potential use of bovine models to further understanding of human PNPLA3 and NAFLD.

## Materials and methods

All animal use and handling protocols were approved by the University of Wisconsin-Madison College of Agricultural and Life Sciences Animal Care and Use Committee and were carried out in accordance with relevant guidelines and regulations. Materials and methods presented follow the ARRIVE guidelines.

### Isolation and cell culture

Primary bovine hepatocytes were isolated from 4 Holstein bull calves (5 ± 2 days; calf as biological replicate) as described previously^12^ and all hepatocyte isolation preparations were used in this experiment (n = 4). The caudate process was excised and perfused using collagenase at 37° C and hepatocytes were isolated as previously described^51–53^. Cells were plated on 35-mm tissue treated dishes (Eppendorf, Hauppauge, NY) in sterile Dulbecco’s Modified Eagle’s Medium (D2902, Sigma-Aldrich, St. Louis, MO) with added cell culture grade HEPES and sodium bicarbonate (DMEM; Sigma-Aldrich) supplemented with 20% fetal bovine serum and 1% antibiotic, antimycotic solution (Sigma-Aldrich) at a density of approximately 2 million cells per dish. Media was refreshed with sterile DMEM supplemented with 10% fetal bovine serum and 1% antibiotic, antimycotic solution 4 h after initial plating. Cells were maintained in monolayer cultures for 24 h and were at least 80% confluent.

### Fatty acid treatments

Wells were randomly assigned to treatment in triplicate. Treatment media comprised of sterile DMEM with the addition of 1% bovine serum albumin (BSA; EMD Millipore, Burlington, MA) and 1% antibiotic, antimycotic solution plus either individual FA (iRECOV) or FA mixtures. Cell culture grade C14:0, C16:0, C18:0, C18:1 n-9, C18:2 n-6, C20:5 n-3, and C22:6 n-3 were independently bound to BSA (9% solution) to achieve individual 8 mM stock FA solutions as described previously^54^. Two FA mixtures were comprised of varying percentages of each stock FA (Table 1) to represent circulating serum FA in vivo^55^ reflective of two distinct postpartum periods (1) accumulation of liver lipids (ACCUM) and (2) recovery from fatty liver (RECOV). Cells were treated with 0.25, 0.5, 0.75, or 1 mM of ACCUM or RECOV, respectively.

### RNA isolation and RT-qPCR analysis

For genes with known strong correlations between expression and protein abundance^50,56–58^, only gene expression was quantified. An unabridged version of RNA isolation and real-time qPCR (RT-qPCR) methods has been published previously^59^. As described therein, cells were harvested in TRIzol reagent (Invitrogen, Carlsbad, CA), RNA extracted using a phenol–chloroform extraction^60^ (Life Technologies), and RNA pooled and purified using the Aurum Total RNA 96 Kit^61^ (732–6800; Bio-Rad Laboratories, Hercules, CA). Quantified (Synergy H1 Hybrid Spectrophotometer; BioTek, Winooski, VT) and quality assured (ratio absorbances between 1.9 and 2.1^52,59^) RNA samples were reverse transcribed to cDNA using 0.5 µg RNA and iScript Reverse Transcription Supermix (170–8840; Bio-Rad Laboratories) in a C1000 Touch Thermo Cycler (Bio-Rad Laboratories). Standard curves and controls were as described previously^59^ and cDNA samples were diluted 1:20 for qPCR.

Expression of genes *ChREBP*, *CPT1A*, *CPT2*, *FASN*, *FOXO1*, *LXRA*, *LXRB*, *PGC1A*, *PNPLA3*, *PPARA*, *SIRT1*, *SIRT3*, *SREBP1c*, and *UCP2* was quantified using RT-qPCR in a CFX-384 Real-Time System (Bio-Rad Laboratories, Hercules, CA) with SsoAdvanced SYBR (172–5270, Bio-Rad Laboratories). Gene expression of reference genes ribosomal 18S (*18S*), glyceraldehyde 3-phosphate dehydrogenase (*GAPDH*), ribosomal protein L32 (*RPL32*), and ribosomal protein S9 (*RPS9*) were quantified as described above and the stability of reference gene normalization using the geometric mean of all four reference genes (*M* = 0.06) instead of a single gene or other gene combination (*M* ≥ 0.09) was confirmed via NormFinder^62^. All primers (Table 3) were optimized and either validated previously or verified within as the single product using melt-peak analysis. Standards, controls, and samples were amplified in triplicate for all genes as follows: 1 cycle at 95° C for 3 min, 45 cycles of 95° C for 15 s and 55° C for 5 s, then a melt curve from 65° C to 95° C at increasing increments of 0.5° C for 3 s. Primers for *PNPLA3*, *SIRT3*, and *SREBP1c* used annealing temperatures of 55.3, 54.3, and 58.4° C instead of 55° C, respectively.

**Table 3:** Primers used for real time-quantitative PCR organized by function: reference, fatty acid (FA)-mediated, and transcription factor (TFs) genes.

Gene^1^	Accession no.	Position^2^	Sequence (5ʹ–3ʹ)	Source
**References**				
*18S*	NR_036642.1	F	ACCCATTCGAACGTCTGCCCTATT	^52^
		R	TCCTTGGATGTGGTAGCCGTTTCT	
*GAPDH*	NM_001034034.2	F	AAGGTCGGAGTGAACGGATTC	^65^
		R	ATGGCGACGATGTCCACTTT	
*RPL32*	NM_001034783.2	F	AGACCCCTCGTGAAGCCTAA	^65^
		R	CCGCCAGTTCCGCTTGATTT	
*RPS9*	NM_001101152.2	F	CCTCGACCAAGAGCTGAA	^53^
		R	CCTCCAGACCTCACGTTT	
**FA-mediated**				
*CPT1A*	NM_001304989.1	F	TTCGCGATGGACTTGCTGTA	^35^
		R	TTTCCTCCCGGTCCAGTTTG	
*CPT2*	NM_001045889.1	F	TGAACATCCTCTCCATCTGG	^39^
		R	GGTCAACAGCAACTACTACG	
*FASN*	NM_001012669.1	F	CAGCTTTGTGTTGGCAGAGAAG	^39^
		R	AGCGAGCTGTCCAGGTTGAC	
*PNPLA3*	XM_005207459.4	F	ACGAAGGGTTCACCAAGCTC	^12^
		R	GCATTAACAGCGACCGGAAC	
*UCP2*	NM_001033611.2	F	ACCTAACACAGCCGGTCTC	Verified within
		R	GCAACCTCCCAGGAGATGAA	
**TFs**				
*ChREBP*	TC350579	F	GCTGCAGCATGGCAATGTACT	^66^
		R	GGAGCAGAAGAGGCGTTCAA	
*FOXO1*	XM_025000053.1	F	AAGCGAGCAAGCAGGCTACA	Verified within
		R	GGACTGCTTCTCTCAGTTCCTG	
*LXRA*	NM_001014861.1	F	CATGCCTACGTCTCCATCCA	^66^
		R	TCACCAGTTTCATCAGCATCCT	
*LXRB*	NM_001014883.1	F	TGCAGCTCGGTCGTGAAG	^66^
		R	CAGCAGCATGATCTCGATGGT	
*PGC1A*	NM_177945.3	F	CAGAAGGCAATTGAAGAGCG	Verified within
		R	CCTCAGTTCTGTCCGTGTTGT	
*PPARA*	NM_001034036.1	F	ACAAAGCCTCTGGCTACCAC	^31^
		R	AGCTTCAGCCGAATCGTTCT	
*SIRT1*	NM_001192980.3	F	AAGACCTCGGATAGGTCCAT	Verified within
		R	GAATTGTTCGAGGATCTGTGCC	
*SIRT3*	XM_005200933.4	F	CAACCTGGAGAAATACCGTCTT	^67^
		R	CAGTCCTTTTTCCTTCAGCAG	
*SREBP1c*	NM_001113302.1	F	CGACACCACCAGCATCAACCACG	^68^
		R	GCAGCCCATTCATCAGCCAGACC	

### Protein isolation and Western Blot analysis

For genes subject to translational regulation, protein abundance was quantified^28,29^. Molecular grade ethanol was added to the phenol-intermediate phase for precipitation of DNA and tubes were centrifuged at 2000×*g* for 5 min at 4 °C and supernatant saved for protein isolation via ethanol addition per the manufacturer’s protocol (Life Technologies). The resulting protein pellet was re-suspended in a modified lysis buffer^63^ with addition of Halt Protease Inhibitor Cocktail (78445; Pierce BioTechnology, ThermoFisher, Rockford, IL) and then warmed at 55 °C until the pellet solubilized as described previously^63^. Samples were centrifuged at 10,000×*g* for 10 min at 4 °C to remove impurities (Life Technologies), and supernatants collected and pooled across technical replicates. As done previously^12^, a protein pool comprised of equal volumes of samples was created as a quality control standard for downstream analysis via Western Blot, herein referred to as the pool.

Protein concentration of samples and the pool was determined by BCA assay per the manufacturer’s protocol (23227, Pierce, ThermoFisher, Rockford, IL). Any samples that did not fall within the standard curve were concentrated using molecular weight concentrators following the manufacturer’s protocol (88502; Pierce, ThermoFisher, Rockford, IL) and samples re-analyzed via BCA assay to determine concentration with coefficient of variations never exceeding 10%.

Samples were prepared for Western Blot analysis as previously described^12,64^. All proteins of interest were probed on Western Blots loaded with 25 µg of protein per lane and were heated at 37 °C for 30 min, except for ChREBP which was analyzed on Western Blots loaded with 50 µg of protein per lane and were heated at 100 °C for 5 min. After blocking, blots were probed with primary antibody for 1 h (ABHD5, ab59488, Abcam, Cambridge, MA; PHSL, 4139S, Cell Signaling Technologies, Danvers, MA; PLIN and PPLIN, AB10200, EMD Millipore Sigma, Darmstadt, Germany; PNPLA3, ab81874, Abcam; SREBP1c, LSB-93, LS-Bio, Seattle, WA) or overnight at 4 °C (ATGL, ab99532, Abcam; ChREBP, sc-515922, Santa Cruz Biotechnologies; HSL; 4107S, Cell Signaling Technologies; SREBP1c, LS-93; Life Science Biology). Before blocking and probing for ChREBP abundance, blots were blocked with an endogenous biotin blocking kit (E-21390; Thermo Fisher Scientific) for 20 min for each reagent and diluted in extra-pure water.

### Hepatocyte imaging of PNPLA3

Bovine neonatal hepatocytes were stained with DAPI (MP01306; Invitrogen, ThermoFisher Scientific) and Alexa Fluor 488 Phalloidin (A12379; Invitrogen, ThermoFisher Scientific) for nuclei and F-actin staining, respectively, following the manufacturer’s protocols (Invitrogen, ThermoFisher Scientific). Cells were then exposed to incubation with the primary antibody used for Western Blot analysis for PNPLA3 but at a 1:400 dilution (ab81874, Abcam). After primary incubation overnight at room temperature, a secondary anti-rabbit Cy3 antibody (711-165-152, Jackson Research Laboratories, West Grove, PA) was administered at 1:100 for a 2 h incubation period. Images were taken on each respective channel, the three images merged, and the final image was altered minimally to reduce background noise.

### Statistical analysis

Data was checked for normality using PROC UNIVARIATE in SAS 9.4 (SAS Institute Inc., Cary, NC) and transformed, when necessary, based on Shapiro-Wilks. Normality was achieved using root transformation, or square root of the multiplicative inverse, and the appropriate transformation determined and applied on an individual gene or protein basis. Data was analyzed using PROC MIXED (SAS 9.4). Separate mixed models were built for FA mixture treatments and iRECOV treatments. For FA mixture treatments, models contained the fixed effect of FA mixture treatment, concentration, their interaction, and random effect of calf. For iRECOV treatments, models contained the fixed effect of individual FA and random effect of calf. Significance was declared at *P* ≤ 0.05 and tendencies at 0.05 < *P* ≤ 0.10. If a main effect of FA mixture, concentration, or the interaction for FA mixture and concentration treatments for either gene expression or protein abundance was *P* ≤ 0.15, quadratic relationships were explored. When the interaction of FA mixture × concentration was significant, means were separated by Tukey–Kramer adjustment. All data reported are least square means ± standard error of the mean (LSM ± SEM).
